# Relationship Between Gardening and Stress on Older Adult Physical and Mental Health

**DOI:** 10.1093/geroni/igaf122.3074

**Published:** 2025-12-31

**Authors:** Laurel Mertz, Jennifer Smith

**Affiliations:** Mather Institute, Evanston, Illinois, United States; Mather Institute, Evanston, Illinois, United States

## Abstract

The act of gardening has significant potential to improve successful aging among older adults, including through improved physical health factors such as better flexibility, blood pressure, and cholesterol. Beyond physical health, gardening may also enhance older adults’ ability to age well through improved socialization, quality of life, cognition, and mental health. This study aimed to extend upon previous research by evaluating the frequency of gardening among older adults and exploring how gardening may attenuate the relationship between stress and health. A total of 3,293 older adults, ages 55 and better, were recruited via an online research panel (mean age = 65.6 years, 52.9% female, 69.6% White). Participants completed a survey assessing frequency of gardening, stress levels, and physical and mental health. Descriptive and independent t-test analyses revealed that 61.6% of older adults reported gardening at least sometimes, and that older adult women gardened significantly more frequently than older adult men. Multiple regression analyses controlling for gender, income, and age indicated that engaging in more frequent gardening attenuated the negative relationship between stress and mental health, but not physical health. That is, higher levels of stress were related to worsened mental health, but this relationship was weaker for those who gardened more frequently. These findings provide evidence that gardening may be effective in protecting against the negative impact of stress on older adults’ mental health. Given the potential protective factor of gardening, future research should explore ways to address barriers to gardening in older adults, especially older adult men.

